# Early detection of small volume stroke and thromboembolic sources with computed tomography: Rationale and design of the ENCLOSE study

**DOI:** 10.1177/2396987320966420

**Published:** 2020-10-23

**Authors:** Frans Kauw, Fasco van Ommen, Edwin Bennink, Maarten J Cramer, L Jaap Kappelle, Richard AP Takx, Birgitta K Velthuis, Max A Viergever, H Wouter van Es, Wouter J Schonewille, Jonathan M Coutinho, Charles BLM Majoie, Henk A Marquering, Hugo WAM de Jong, Jan W Dankbaar

**Affiliations:** 1 Department of Radiology, University Medical Center Utrecht, Utrecht University, Utrecht, The Netherlands; 2 Image Sciences Institute, University Medical Center Utrecht, Utrecht, The Netherlands; 2 Department of Neurology and Neurosurgery, Brain Center, University Medical Center Utrecht, Utrecht, Utrecht University, The Netherlands; 4 Department of Cardiology, University Medical Center Utrecht, Utrecht University, Utrecht, The Netherlands; 5 Department of Radiology, St. Antonius Hospital, Nieuwegein, The Netherlands; 6 Department of Neurology, St. Antonius Hospital, Nieuwegein, The Netherlands; 7 Department of Neurology, Amsterdam UMC, Amsterdam, The Netherlands; 8 Department of Radiology and Nuclear Medicine, Amsterdam UMC, Amsterdam, The Netherlands; 9 Department of Biomedical Engineering and Physics, Amsterdam UMC, Amsterdam, The Netherlands

**Keywords:** Acute ischemic stroke, computed tomography, cardiac thrombus, recurrent stroke, prediction model, magnetic resonance imaging, lacunar stroke, detection

## Abstract

**Background:**

Computed tomography is the most frequently used imaging modality in acute stroke imaging protocols. Detection of small volume infarcts in the brain and cardioembolic sources of stroke is difficult with current computed tomography protocols. Furthermore, the role of computed tomography findings to predict recurrent ischemic stroke is unclear. With ENCLOSE, we aim to improve (1) the detection of small volume infarcts with thin slice computed tomography perfusion (CTP) images and thromboembolic source with cardiac computed tomography techniques in the acute stage of ischemic stroke and (2) prediction of recurrent ischemic stroke with computed tomography-derived predictors.

Methods/design: ENCLOSE is a prospective multicenter observational cohort study, which will be conducted in three Dutch stroke centers (ClinicalTrials.gov Identifier: NCT04019483). Patients (≥18 years) with suspected acute ischemic stroke who undergo computed tomography imaging within 9 h after symptom onset are eligible. Computed tomography imaging includes non-contrast CT, CTP, and computed tomography angiography (CTA) from base of the heart to the top of the brain. Dual-energy CT data will be acquired when possible, and thin-slice CTP reconstructions will be obtained in addition to standard 5 mm CTP data. CTP data will be processed with commercially available software and locally developed model-based methods. The post-processed thin-slice CTP images will be compared to the standard CTP images and to magnetic resonance diffusion-weighted imaging performed within 48 h after admission. Detection of cardioembolic sources of stroke will be evaluated on the CTA images. Recurrence will be evaluated 90 days and two years after the index event. The added value of imaging findings to prognostic models for recurrent ischemic stroke will be evaluated.

**Conclusion:**

The aim of ENCLOSE is to improve early detection of small volume stroke and thromboembolic sources and to improve prediction of recurrence in patients with acute ischemic stroke.

## Background

Acute ischemic stroke (AIS) is a medical emergency associated with high rates of mortality and morbidity.^1^ Rapid imaging is key in the acute stroke setting as detection of ischemia and cause of the stroke is essential for treatment decisions. Currently, non-contrast computed tomography (NCCT), CT perfusion (CTP), and CT angiography (CTA) are routinely performed as part of most acute stroke imaging protocols.^2^ NCCT is used to exclude hemorrhagic stroke and to identify early signs of ischemia. CTA is required to identify the site and extent of arterial occlusion with the aim to determine eligibility for endovascular treatment (EVT). In addition, evaluation of collateral circulation on CTA helps to guide treatment strategies and predict patient outcomes.^3–5^ Important causes of stroke such as large artery atherosclerosis, dissection, or cardioembolism can also be identified with CTA. Perfusion maps generated from CTP source data may guide decision-making for intravenous thrombolysis (IVT) or EVT in the extended window and in patients with unknown time of onset of stroke (e.g. wake-up stroke) as eligibility strongly depends on infarct core and penumbra volumes.^6–8^ In addition, small perfusion defects can pinpoint more distal arterial occlusions that are difficult to detect on CTA. Also, time-invariant CTA, which is derived from the CTP source data, enables accurate assessment of the thrombus extent and the collateral status.^9^

CT imaging has certain advantages over magnetic resonance imaging (MRI) in evaluating patients with suspected AIS, such as 24/7 availability, speed, and lower costs. CTP has high sensitivity (80%) and very high specificity (95%) for infarct detection and has added value to NCCT and CTA for posterior infarct detection.^10^,^11^ However, not all clinical CT scanners have full-brain CTP coverage, which may result in false-negative findings.^10^ In addition, high noise levels and reconstructions with low spatial resolution limit the ability of CTP to identify small volume infarcts.^12^,^13^ In this study, small volume stroke was defined as a clinical diagnosis of AIS or transient ischemic attack with one of the following imaging findings: no occlusion visible on the admission CTA scan; an occlusion distal to the A2 segment of the anterior cerebral artery; an occlusion distal to the M1–M2 bifurcation of the middle cerebral artery bifurcation; an occlusion of the posterior circulation without involvement of the basilar artery.

Stroke etiology has been established as an important predictor of recurrent ischemic stroke.^14^ Thromboembolic sources of stroke include large artery atherosclerosis, dissection, and cardioembolism. In about a quarter of patients with ischemic stroke, cardioembolism is the cause of the stroke, which requires specific treatment to prevent recurrent events. The cause of the stroke remains unclear in 20–40% of the patients after routine work-up.^15–19^ An important cause of this so-called cryptogenic stroke is paroxysmal atrial fibrillation, which often remains undetected in the early stroke phase despite continuous ECG monitoring. Paroxysmal atrial fibrillation may be detected in a later phase, but the treatment-delay causes a prolonged time window of increased recurrence risk.^20^ Transthoracic echocardiography is the standard imaging modality to detect cardiac thrombus, but has limited sensitivity for this purpose depending on patient habitus and the available imaging window. Transesophageal echocardiography has a higher accuracy, but is only performed on strict indication because it is an invasive procedure, uncomfortable for the patient, and often requires light sedation. In case of delay in cardiac imaging, the culprit thrombus may have resolved. Cardiac CTA has been proposed as a fast and noninvasive alternative method for depicting cardiac thrombus.^21–23^ Non-ECG-triggered cardiac CTA can be performed in the acute stroke setting by extending the stroke CTA to include the heart with minor adjustments to the scan parameters so that the amount of contrast agent, the radiation dose, and the acquisition duration do not increase. Alternatively, ECG-triggered cardiac CTA can be performed separately in the same stroke-imaging session or soon after treatment. Cardiac CTA is performed as part of routine care in the participating centers of ENCLOSE in patients with suspected AIS. One center will use dual-energy CT, which allows for increased iodine contrast and differentiating between iodine, calcifications, slow flow, and motion artifacts. This may facilitate identification of cardiac thrombus and characterization of large artery atherosclerosis. The first goal of ENCLOSE is to improve detection of small volume infarcts and thromboembolic sources with state-of-the-art CT techniques.

The two-year recurrence rate has been estimated to be 12% in patients with AIS.^24^ Clinical prediction models have been developed for predicting recurrence, but heterogeneity is high.^25^ CT-derived predictors have been identified as prognostic for clinical outcome after AIS, but not yet for recurrences.^14^ The second goal of ENCLOSE is to prospectively evaluate the added value of CT-derived predictors for predicting recurrent ischemic stroke. If applicable, MRI-derived predictors will be evaluated additionally.

## Methods/design

### Study design

**Figure 1.**
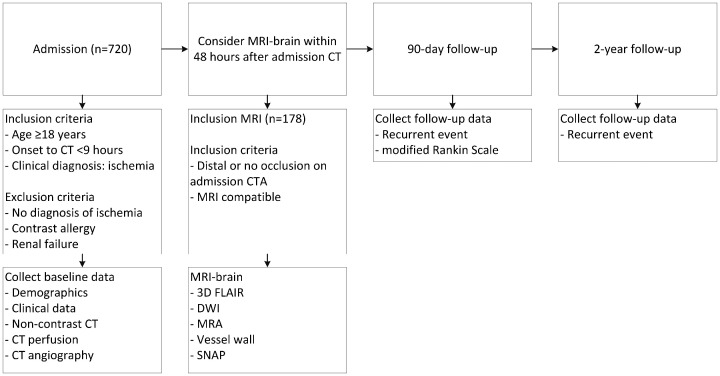
Flowchart of patient selection, study procedures, and outcome evaluations. Distal occlusion on admission CTA is defined as an occlusion distal to the A2 segment of the anterior cerebral artery, distal to the M1–M2 bifurcation of the middle cerebral artery, or an occlusion of the posterior circulation excluding basilar and vertebral artery occlusions. CT: computed tomography; MRI: magnetic resonance imaging; A2: A2 segment of anterior cerebral artery; M1–M2: junction between M1 and M2 segments of the middle cerebral artery; CTA: computed tomography angiography; FLAIR: fluid-attenuated inversion recovery; DWI: diffusion-weighted imaging; MRA: magnetic resonance angiography; SNAP: simultaneous non-contrast angiography and intraplaque hemorrhage.

ENCLOSE (ClinicalTrials.gov Identifier: NCT04019483) is a prospective multicenter observational cohort study, which will be conducted in three Dutch stroke centers: the University Medical Center (UMC) Utrecht, the Amsterdam UMC—location Academic Medical Center (AMC) and the St. Antonius Hospital—location Nieuwegein. Patient selection, study procedures, and outcome evaluations are described in Figure 1. We will include 720 patients with suspected AIS. At presentation, patients undergo clinical evaluation, laboratory testing, and CT imaging according to local stroke protocols. The local imaging protocol for stroke includes NCCT, CTP, and CTA. Two centers will perform non-ECG-triggered CTA from the base of the heart up to the top of the head; one center will perform a non-ECG-triggered CTA from the aortic arch to the head and a separate ECG-triggered cardiac CTA. Patients with a suspected small volume infarct will be asked to undergo an additional brain-MRI within 48 h after hospital admission. The included patients will be followed for two years to identify recurrent ischemic strokes and to evaluate 90-day clinical outcome.

### Inclusion and exclusion criteria

Patients will be included if the following criteria are met: age of 18 years or older, time from symptom onset, or last seen well until admission CT is less than 9 h,^26^ clinical diagnosis of AIS or transient ischemic attack (TIA), and informed consent from patient or legal representative. Patients who awake with stroke symptoms can only be included if they went to sleep without any stroke symptoms, and the time from going to sleep until imaging is less than 9 h. To participate in the MRI-part of this study, the patient also has to meet the following criteria: no occlusion visible on the admission of CTA scan or an occlusion distal to the A2 segment of the anterior cerebral artery, distal to the M1–M2 bifurcation of the middle cerebral artery bifurcation or an occlusion of the posterior circulation without involvement of the basilar artery, and no contraindications for undergoing MRI. Patients with another diagnosis such as intracerebral hemorrhage, subarachnoid hemorrhage or tumor, or patients with known contrast allergy or renal failure will be excluded from the study.

### Baseline data

#### Clinical data

The following baseline characteristics will be collected: age, sex, time of symptom onset or last seen well in case of a wake-up stroke or other strokes with unknown time of onset, National Institutes of Health Stroke Scale (NIHSS), and the Trial of Org 10172 in Acute Stroke Treatment (TOAST) classification. The following cardiovascular risk factors will be recorded: history of stroke, coronary heart disease, atrial fibrillation, congestive heart failure, valvular disease, peripheral vascular disease, active cancer, hypertension, dyslipidemia, diabetes mellitus, smoking, and medication use. Biometry data include length and weight. Furthermore, therapeutic interventions such as intravenous administration of tissue-type plasminogen activator (tPA), endovascular stroke treatment, and carotid endarterectomy will be recorded. Laboratory data include levels of hemoglobin, thrombocytes, glucose, HbA1c, creatinine, C-reactive protein, lipids, and international normalized ratio. Medication use on hospital discharge will be collected as well.

#### CT imaging

##### Acquisition

Patients with suspected ischemic stroke will undergo NCCT, CTP, and CTA. One institution will use a dual-energy CT scanner as part of standard care. The local stroke imaging protocols from the three participating hospitals will be used. Contrast and flow parameters for CTP and CTA are shown in Table 1. The acquisition and reconstruction parameters for the different CT scanners are shown in Tables 2 and 3.

**Table 1.** Contrast parameters for CT perfusion and CT angiography acquisition.

Center	Scanner	Contrast	CTP				CTA			
			Bolus (ml)	Flow (ml/s)	Saline (ml)	Flow (ml/s)	Bolus (ml)	Flow (ml/s)	Saline (ml)	Flow (ml/s)
UMC Utrecht	Philips Brilliance iCT-256	Ultravist-300 mg/ml	40	6	40	6	65	6	40	6
	Philips IQon spectral CT	Ultravist-300 mg/ml	50	6	40	6	65	6	40	6
Amsterdam UMC	Siemens Force	Iomeron 300 mg/ml	35	6	40	6	50	6	40	6
	Siemens AS+	Iomeron 300 mg/ml	35	6	40	6	50	6	40	6
St. Antonius Hospital	64-Brilliance	Xenetix 300 mg/ml	40	5	50	4	55	4	40	4
	256-Brilliance iCT Philips	Xenetix 300 mg/ml	40	5	50	4	55	4	40	4

**Table 2.** Acquisition and reconstruction parameters of the CT perfusion stroke protocols in the participating centers.

Center	Scanner	Scan type	Peak voltage (kVp)	Exposure (mAs)	Beam collimation (mm)	Slice thickness/increment (mm)	Reconstruction (level), kernel	Matrix
UMC Utrecht	Philips Brilliance iCT-256	Axial	80	150	128 × 0.625	5.0/5.0 of 1.0/0.8	iDose (5), UB	512 × 512
	Philips IQon spectral CT	Jog Mode	120	35	64 × 0.625	5.0/5.0 of 0.9/0.7	iDose (5), UB	512 × 512
Amsterdam UMC	Siemens Force	Axial	80	84	192 × 0.6	1.0/0.7	Admire (1), Bv36	512 × 512
	Siemens AS+	Axial	80	200	32 × 0.6	5.0/5.9	Saffire (1), H20f	512 × 512
St. Antonius Hospital	64-Brilliance	Axial	80	125	64 × 0.625	5.0/5.288	iDose (3), B	512 × 512
	256-Brilliance iCT Philips	Axial	80	125	128 × 0.625	5.0/5.0	iDose (5), B	512 × 512

**Table 3.** Acquisition and reconstruction parameters of the CT angiography stroke protocols in the participating centers.

Center	Scanner	Scan type	Peak voltage (kVp)	Exposure (mAs)	Pitch	Beam collimation (mm)	Slice thickness/increment (mm)	Reconstruction (level), kernel	Matrix
UMC Utrecht	Philips Brilliance iCT-256	Helical	100	AEC	0.914	128 × 0.625	3.0/3.0 or 0.9/0.45	iDose (3), B	512 × 512
	Philips IQon spectral CT	Helical	120	AEC	0.609	64 × 0.625	5.0/5.0 or 0.9/0.7	iDose (5), B	512 × 512
Amsterdam UMC	Siemens Force	Helical	100	200	0.55	192 × 0.6	1.0/0.7	Admire (1), Bv36	512 × 512
	Siemens AS+	Helical	100	200	0.55	128 × 0.6	1.0/1.0	Saffire (1), I46f	512 × 512
St. Antonius Hospital	64-Brilliance	Helical	100	230	1.033	64 × 0.625	2.0/1.5	iDose (3), B	512 × 512
	256-Brilliance iCT Philips	Helical	100	230	0.763	128 × 0.625	2.0/1.5	iDose (3), B	512 × 512

AEC means automatic exposure control.

##### Analysis

The acquired admission CT data are stored on an online secured server in the University Medical Center Utrecht. Standard post-processing of the data with 5-mm CTP reconstructions will be performed with Intellispace software (Philips Healthcare, Best, The Netherlands). In addition, the thin-slice CTP source data will also be reconstructed with iterative reconstruction and processed with in-house developed CTP software to enable detection of small volume strokes on admission CTP.^27^ If the subject is scanned on the Philips IQon Spectral CT, the dual-energy CTP dataset will be used to reconstruct images including virtual monoenergetic images, virtual non-contrast images, and iodine maps. The obtained perfusion maps will be evaluated for the presence and location of a focal perfusion deficit matching a part of a cerebral artery flow-territory. Fat and calcium volume of internal carotid artery atherosclerotic plaque will be determined on dual-energy CTA. The admission non-gated cardiac CTA will be evaluated for the presence of thrombus, left atrial size and atrial appendage morphology, patent foramen ovale or other septal defects, signs of previous myocardial ischemia or non-ischemic cardiomyopathy, aneurysm, valvular abnormalities, and signs of endocarditis. Imaging assessments will be done by experienced observers, who will be blinded to clinical data.

### Follow-up data

#### Cardiac imaging

In case follow-up imaging of the heart (e.g. transthoracic or transesophageal echocardiography, CT, or MRI) is performed, the same variables will be evaluated as for baseline cardiac CTA. Other remarkable findings will be noted. Findings from continuous ECG monitoring or Holter ECG will also be recorded.

#### MR imaging

Patients who fulfill the selection criteria for the MRI-part of this study will undergo MRI of the brain and neck within 48 h after presentation. MRI findings will serve as the gold standard for the detection of cerebral ischemia. The MRI protocol will include diffusion-weighted imaging (DWI) and 3D fluid attenuated inversion recovery (FLAIR) sequences of the brain, and, if feasible, MR angiography (MRA) of the neck, vessel wall imaging of the intracranial arteries, and simultaneous non-contrast angiography and intraplaque hemorrhage (SNAP) sequences of the extracranial carotid arteries.^28^ Participants will be scanned on 3T MRI scanners from different vendors. If 3T MRI is not available for logistic reasons, FLAIR and DWI sequences can be acquired on 1.5-T MRI scanners instead. In case an MRI is performed as part of routine clinical care within 48 h after CT imaging, the acquired FLAIR and DWI images will be used instead of performing an additional study MRI.

#### Recurrence and clinical outcome

As part of routine care, patients will be contacted for follow-up evaluation at 90 days in the outpatient clinic or by telephone by stroke nurses, who are trained and certified to evaluate the modified Rankin scale (mRS). The 90-day clinical outcome of the patient will be evaluated with the mRS. A Dutch-structured questionnaire, which was based on previous studies, will be used to assess the mRS by telephone.^29^,^30^ In addition, patients will be asked for a second follow-up evaluation by telephone after two years. At two years, the patients will be asked whether a recurrent stroke has occurred and, if applicable, when and where the patient was treated for the recurrence. A discharge summary on the recurrent event will be requested from the hospital or general practitioner.

### Data entry and monitoring

Clinical data and imaging findings are registered with electronic case report forms in an online secured database (OpenClinica, LLC, Waltham, USA). Patient names and identification numbers will be replaced by a study code. To assess the quality and validity of the research data, independent and qualified monitors will be appointed to monitor the study in the participating centers.

### Statistical analysis

#### Ischemia detection

##### Sample size

Over 30,000 people suffer from an ischemic stroke in the Netherlands each year.^31^ About 30% of them will have an intracranial large artery occlusion, and 70% will have a minor stroke or TIA. As a result, 500 of the 720 subjects are possible candidates for small volume infarct detection. Assuming that at least half of the 500 patients will give informed consent for the extra follow-up MRI and do not have contra-indications for undergoing MRI, we should have 250 potential patients for the part of the study that concerns small volume stroke detection. Based on the historical cohort (Dutch Acute Stroke Study: 2009–2013, METC number 08–373), we expect to improve the sensitivity of CTP for small volume stroke detection from 0.45 to at least 0.75, which approaches the sensitivity of MRI (0.88–1.00).^32^ With a minimal acceptable lower 95% confidence level of 0.6 for the increased sensitivity, we will need about 107 MRI subjects.^33^ We expect that about 60% of patients with suspected stroke, and no large artery occlusion on admission will have an infarct on follow-up imaging.^34^ This means that we need to include at least 178 patients for the MRI part of this study.

### Diagnostic value

The diagnostic properties to detect cerebral ischemia of both the standard CTP and the high-resolution CTP images will be compared to the reference standard (DWI). The DWI images will be evaluated by an experienced neuroradiologist. Sensitivity, specificity, positive predictive value, and negative predictive value with 95% confidence intervals will be calculated. We hypothesize that the high-resolution CTP will have superior sensitivity compared to the standard CTP for the detection of ischemia. The diagnostic values will be compared between the CTP protocols with the McNemar test and with receiver-operating characteristic (ROC) curves. A p-value lower than 0.05 will be considered significant.

#### Recurrent stroke prediction

##### Sample size

For this study, a prediction model containing a maximum of six predictors will be developed for recurrent stroke. Therefore, at least 60 patients with a recurrent stroke are needed according to statistical guidelines.^35^ Ischemic stroke is estimated to recur in approximately 10% of the included patients.^36^ When accounting for a loss to follow-up rate of 10%, the required sample size will be approximately 650. Based on a historical stroke cohort (Dutch Acute Stroke Study: 2009–2013, METC number 08–373), we expect a participation rate of 90%.^37^ Thus, a minimum of 720 patients need to be asked for study participation. In a period of 36 months, at least 720 patients with suspected ischemic stroke will visit the Amsterdam UMC—location AMC, the St. Antonius hospital—location Nieuwegein and the UMC Utrecht.

##### Candidate predictors

In this study, clinical and imaging predictors of recurrent stroke will be analyzed. Previously identified clinical predictors include cardiovascular risk factors such as higher age, prior stroke, prior myocardial infarction, atrial fibrillation, hypertension, diabetes mellitus, and hyperlipidemia.^25^ Predictors, that can be determined on imaging modalities, include previous cerebral infarcts on NCCT, ASPECTS on CTP, and CTA findings such as occlusion or stenosis, collateral filling in the occluded area, carotid plaque characteristics, and presence of a cardioembolic source. Stroke subtype (TOAST) classification is based on both clinical and imaging data.

##### Model derivation

Complete case analysis will be performed as we expect only few missing values. A multivariable model containing all candidate predictors will be developed. The best predictive variables will be selected with backward selection. Akaike’s information criterion will be used to determine the best model fit. Hazard ratios and 95% confidence intervals will be calculated with Cox proportional hazards models. The proportionality hazards assumption will be checked by visual inspection of the Schoenfeld residual plots. The performance of the final model will be evaluated with measures of discrimination and calibration. Discrimination will be assessed with ROC curves and c-statistics. Calibration will be assessed with calibration plots.

### Ethical considerations

Written informed consent will be acquired from patient or legal representative. The ability of the patient to give informed consent will be evaluated in order to determine whether the patient or the legal representative should be asked for written informed consent. Patients who are incapable of giving informed consent will not be asked to undergo MRI. In case the patient dies before informed consent can be acquired, the patient will be included without obtaining informed consent from his/her legal representative as it is undesirable to burden the legal representative with this request. The ENCLOSE study has been approved by the medical ethics committee of the University Medical Center Utrecht.

## Discussion

CTP has good sensitivity (80%) and specificity (95%) for brain infarct detection.^10^,^11^ However, accurate detection of small volume infarcts is still difficult with CTP because of noise and limited spatial resolution.^38^ In addition, evaluation of the posterior fossa is often hampered by streak artefacts.^39^ In case no stroke is visualized with CT, an MRI is sometimes indicated to ascertain the diagnosis. By improving the detection of small volume stroke by CTP, additional MRI may become unnecessary in the diagnostic pathway, and early detection of these small ischemic strokes may influence acute treatment decisions.

Cardiac CTA may be a fast and noninvasive method for depicting cardiac thrombus in the acute stroke phase. It has some advantages over (delayed) echocardiographic imaging and may facilitate in starting anticoagulant treatment in time. Adding cardiac CTA to the standard stroke imaging protocol will cost an additional 2 s, and the additional radiation exposure will be marginal.

Prediction of recurrences has been subject of multiple studies.^25^ However, developed clinical models for long-term prediction have limited discriminative ability with c-statistics not greater than 0.7.^25^ Addition of imaging predictors to clinical models may improve their predictive value.

For this study, we will not exclude particular subgroups of patients with AIS for reasons of clinical applicability and generalizability. We select on acquisition timing as CT findings such as ischemia may differ between patients who present in an early time window when compared with patients who present in a late time window. Different kinds of CT scanners will be used as multiple centers are involved in this study. Although this can lead to heterogeneity of imaging acquisitions, it enables generalization of the results to other stroke centers.

In conclusion, the ENCLOSE study aims to increase the diagnostic accuracy of CTP for detection of small volume infarcts and diagnostic yield of CTA for cardioembolic sources. Besides, the study aims to improve prognostic models for predicting recurrent ischemic stroke. CT-derived predictors, including predictors derived from early cardiac CTA, will be used to improve prognostication of recurrent ischemic stroke.
